# A Comparison of the Apical Sealing Efficacy Between Guttaflow Bioseal and Mineral Trioxide Aggregate as Root-End Filling Materials: An In Vitro Study

**DOI:** 10.7759/cureus.73498

**Published:** 2024-11-12

**Authors:** Ali B Hseen, Qusay K Nassif, Khetam Maarawi, Radwan A Haffaf, Mayssam Khaddam

**Affiliations:** 1 Department of Endodontics, Tishreen University, Lattakia, SYR; 2 Department of Fixed Prosthodontics, Al-Wadi International University, Homs, SYR; 3 Department of Endodontics, Hama University, Hama, SYR; 4 Department of Orthodontics, Tishreen University, Lattakia, SYR

**Keywords:** apical sealing, endodontics, guttaflow bioseal, mineral trioxide aggregate, mta, ultrasonic

## Abstract

Aim

The objective of this study is to evaluate the efficacy of Guttaflow Bioseal (Coltene/Whaledent, Altstätten, Switzerland) in achieving an apical seal when utilized as a retrograde filling material in comparison to mineral trioxide aggregate (MTA).

Methods

Twenty single-rooted single-canaled human teeth were randomly allocated into two equal groups according to the used retrograde filling materials: Guttaflow Bioseal in group I and MTA in group II. The crowns were sectioned, and the root canals were prepared with rotary files and obturated (single cone technique with a resin-based sealer). Following a 24-hour setting period, apex cutting and ultrasonic retrograde preparation were conducted. Following cavity preparation, the materials were applied into the retrograde cavity (Guttaflow Bioseal in group I and MTA in group II). Both groups were incubated at 37°C with 100% humidity for an additional 24 hours. Subsequently, the teeth were coated with two layers of varnish, leaving the apex exposed by 1 mm. The specimens were then desiccated and immersed in 2% methylene blue dye for 36 hours. Afterwards, the teeth were thoroughly rinsed, dried, and sectioned longitudinally. Dye leakage was examined under optical magnification, and the results were statistically analyzed utilizing the Mann-Whitney U test.

Results

The comparative evaluation revealed no statistically significant difference in microleakage between the two study groups (p-value > 0.05). The apical seal achieved with Guttaflow Bioseal was comparable to that achieved using MTA.

Conclusions

Guttaflow Bioseal demonstrated comparable low microleakage rates and an effective apical sealing capacity similar to that of MTA. Consequently, both materials are deemed suitable for use in retrograde filling applications. Guttaflow Bioseal is a viable option for use as a root-end filling material.

## Introduction

Endodontic therapy is a procedure that aims to limit and remove the infection that occurs in the dental pulp and prevent any further bacterial infection. The usual and conservative way to do endodontic therapy is by making access through the tooth crown. Nonetheless, the intricate anatomy of the root canal system, coupled with the challenges associated with eradicating all bacterial pathogens, can result in treatment failure. In such instances, endodontic retreatment becomes necessary, with non-surgical conservative endodontic retreatment being the preferred initial approach. This includes removing the crown restoration and the root canal filling material, followed by cleaning, reshaping, and proper sealing of the root canal. The advent of advanced endodontic instruments and materials has demonstrated that conservative endodontic retreatment can achieve success rates ranging from 53% to 98% when conducted as a first-line intervention. However, it is important to note that these success rates may be diminished in cases involving teeth with periapical lesions [1,2].

Non-surgical endodontic retreatment can present significant challenges and may result in various complications. These complications include difficulties in accessing the pulp chamber through existing prosthetic or restorative materials, as well as potential perforations and challenges associated with the removal of old obturation materials from the root canal. Such difficulties can compromise the cleaning and shaping of the root canal system [3]. Hence, clinicians might need to perform an apicoectomy to achieve an appropriate seal at the root apex to inhibit the bacterial transition between the root canal and the surrounding tissues. The apicoectomy procedure involves the creation of a full-thickness gingival flap, the excision of surrounding bone to expose the apex of the affected root, resection of the root apex, the preparation of a class I retrograde cavity, and the subsequent placement of a biocompatible retrograde filling material [4].

The success of an apicoectomy is contingent upon the selection of a retrograde filling material that possesses several critical qualities: it must be non-toxic, electrochemically stable, and exhibit high biocompatibility with periradicular tissues. Additionally, the material should demonstrate effective sealing properties and sufficient radiopacity, promote healing, facilitate ease of handling, and remain unaffected by moisture [5]. Several materials have been used as retrograde fillings, such as amalgam, gutta-percha, super ethoxybenzoic acid (EBA), intermediate restorative material, glass ionomer cement (GIC), composite resin, zinc phosphate cement, and mineral trioxide aggregate (MTA). However, it is noteworthy that no single material currently meets all the ideal criteria; thus, the pursuit of an optimal retrograde filling material remains an important area of investigation [6].

Coltene/Whaledent (Altstätten, Switzerland) has recently introduced Guttaflow Bioseal, which is a cold-filling system for root canals and a silicon-based sealant. The formulation of Guttaflow Bioseal comprises several key components, including gutta-percha powder, polydimethylsiloxane, bioactive glass ceramic, platinum catalysis, zirconium dioxide, barium sulfate, microsilver, and color pigments [6]. Multiple studies have demonstrated that Guttaflow Bioseal exhibits superior sealing capability and enhanced biocompatibility in comparison to alternative sealers. This elevated biocompatibility may be attributed to the inclusion of bioactive constituents such as calcium and silicates, alongside the absence of resin-based components [6,7]. Collado-González et al. found that Guttaflow Bioseal demonstrated greater biocompatibility than GuttaFlow 2, MTA Fillapex, and AH Plus when they evaluated the cellular toxicity on the stem cells taken from periodontal ligament [7]. Guttaflow Bioseal uniquely integrates the characteristics of both a sealer and gutta-percha, offering a high sealing ability with a dual-component system packaged in two separate tubes that facilitate a 1:1 mixing ratio. It is characterized by the absence of bubble formation, effective penetration into dentinal tubules and secondary canals, eugenol-free composition, radiopacity, non-shrinkage, and an expansion property. Additionally, it possesses high biocompatibility, is pink in color, provides robust adhesion to dentin, has a working time of five minutes, and has a setting time ranging from 12 to 16 minutes. The primary objective of the current study was to evaluate the efficacy of Guttaflow Bioseal in establishing an apical seal when utilized as a retrograde filling material in comparison to MTA.

## Materials and methods

The study protocol received approval from the Institutional Review Board (IRB) at the Faculty of Dentistry, Tishreen University (Lattakia, Syrian Arab Republic), under approval number 2343\2022. This investigation was designed as an in vitro study utilizing a sample comprised of 20 freshly extracted, single-rooted, single-canal human teeth that were completely intact and devoid of caries, cracks, or deformities. It is important to note that none of the teeth were extracted specifically for this study. The teeth underwent a cleaning process, followed by the removal of periodontal tissues, and were subsequently stored in normal saline at room temperature. Normal saline was refreshed daily until the sample collection was finalized.

Inclusion criteria were as follows: (1) newly extracted lower premolar teeth; (2) teeth exhibiting a single root with a closed apex; (3) absence of carious lesions and cracks; (4) roots that are straight and devoid of any deformities or signs of resorption, whether internal or external.

**Figure 1:**
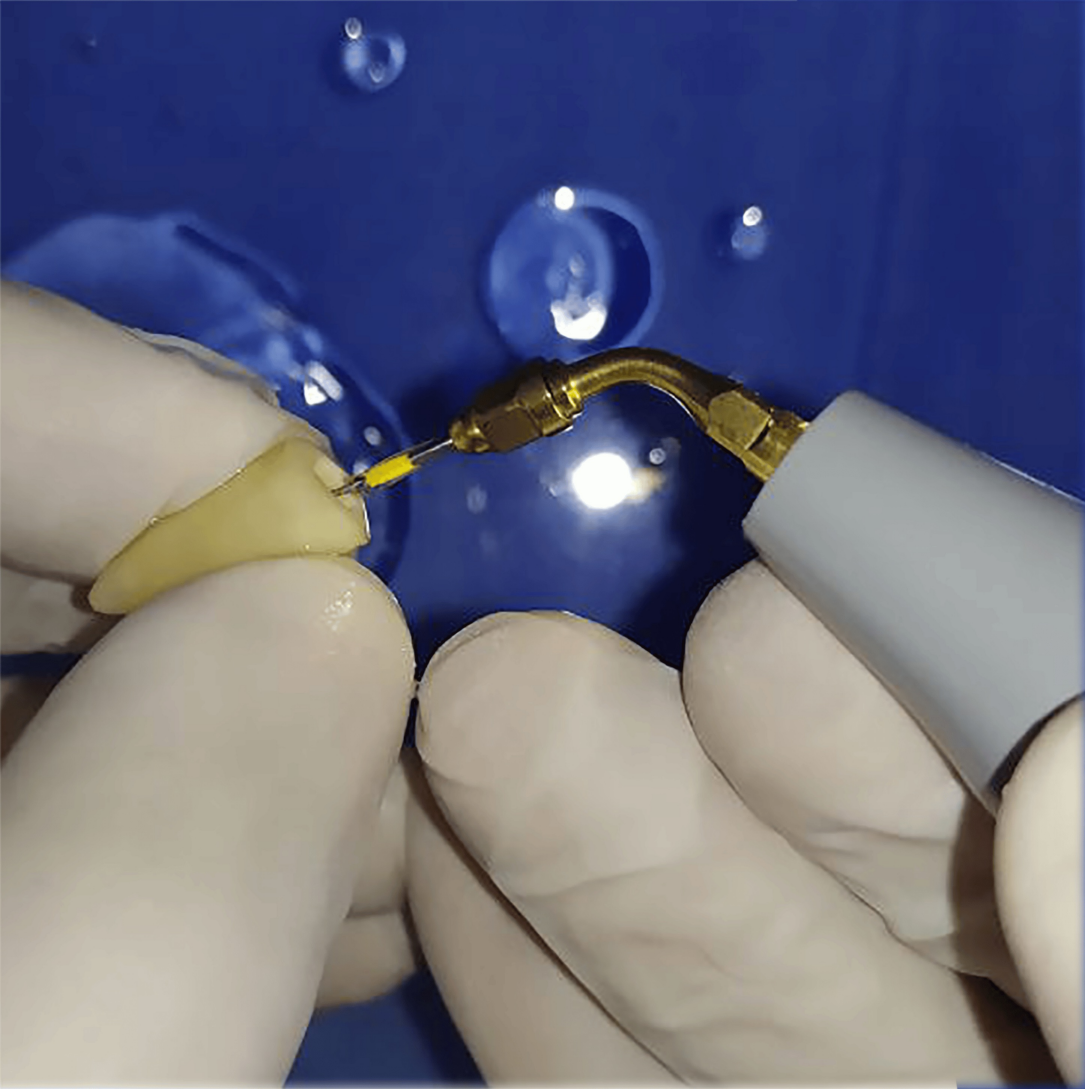
Ultrasonic activation during the root canal preparation.

Teeth were sectioned at the cementoenamel junction, and orifices were accessed using fissure diamond burs. The dental pulp was extirpated, and the working length was established through visual inspection, identifying the tip of a No. 15 file at the root apex, followed by a reduction of 0.5 mm from this measurement. Canals were prepared using endodontic rotary files No. 25 taper 4% (UDG, Changzhou, China). Irrigation was performed using sodium hypochlorite 5.25%, and activation was undertaken using an ultrasonic system (Woodpecker, Guangxi, China) (Figure 1).

**Figure 2:**
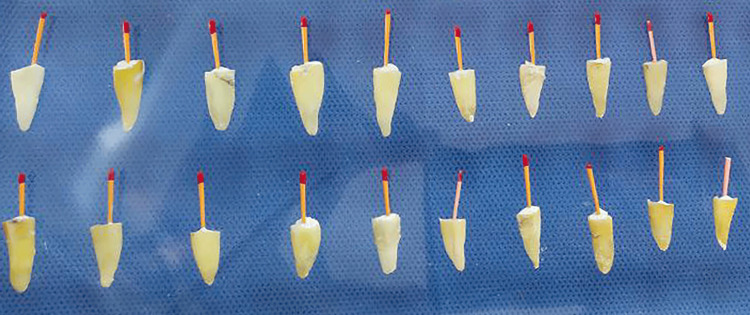
Teeth obturation with single cone technique in the study sample.

Ethylenediaminetetraacetic acid (EDTA) 17% solution (Meta Biomed, Cheongju-si, South Korea) was used for final irrigation. Subsequently, the canals were dried utilizing size #25 taper 4% paper points. Obturation was performed using ADSEAL sealer and gutta percha (Meta Biomed) utilizing the single cone technique (Figure 2).

Crown sealing was conducted utilizing glass ionomer cement (BMS Corporation, Capannoli, Italy), after which the teeth were maintained at room temperature for a duration of 24 hours to allow for proper setting.

**Figure 3:**
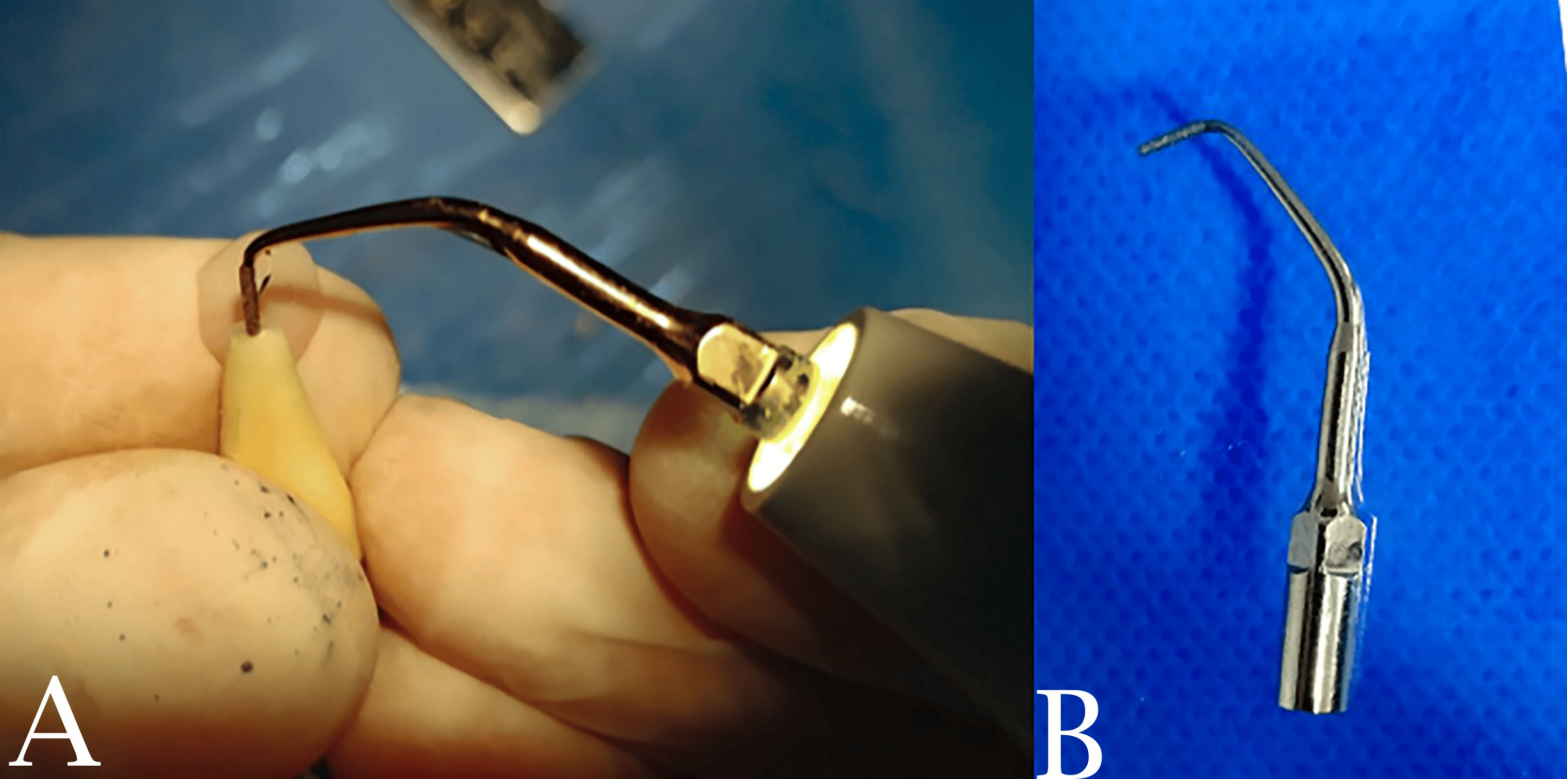
Retrograde cavity preparation using the ultrasonic system. A: Cavity preparation. B: The ultrasonic tip E10D.

Apicoectomy was performed at a 3 mm distance from the apical third of the root at a 90-degree angle using a fissure diamond bur mounted on a high-speed handpiece with water irrigation. The retrograde cavity was performed using the ultrasonic tip E10D (Figure 3). William’s probe was used to ascertain the depth of the retrograde cavity.

**Figure 4:**
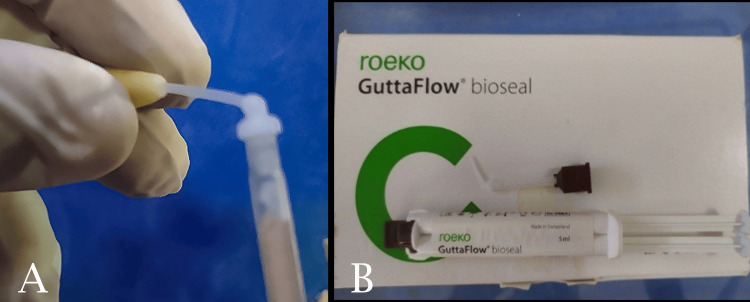
Guttaflow Bioseal application in the retrograde cavity. A: Direct application of Guttaflow Bioseal. B: Guttaflow Bioseal syringe and dispenser.

Following the preparation of cavities, a total of 20 teeth were assigned to two distinct groups (n = 10 each): the Guttaflow Bioseal group and the MTA group. In the Guttaflow Bioseal group, the material was applied directly into the retrograde cavity utilizing specialized mixing tips (Figure 4).

**Figure 5:**
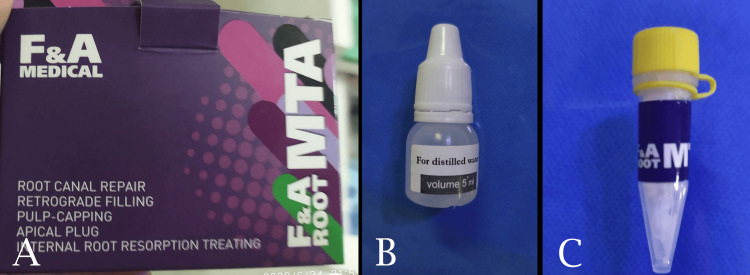
Mineral trioxide aggregate material used in group II. A: The package contains two bottles. B: Distilled water bottle. C: Powder bottle.

In the MTA group, MTA (F&A Medical Corporation, Dubai, UAE) was mixed with distilled water and applied in the retrograde cavity (Figure 5).

**Figure 6:**
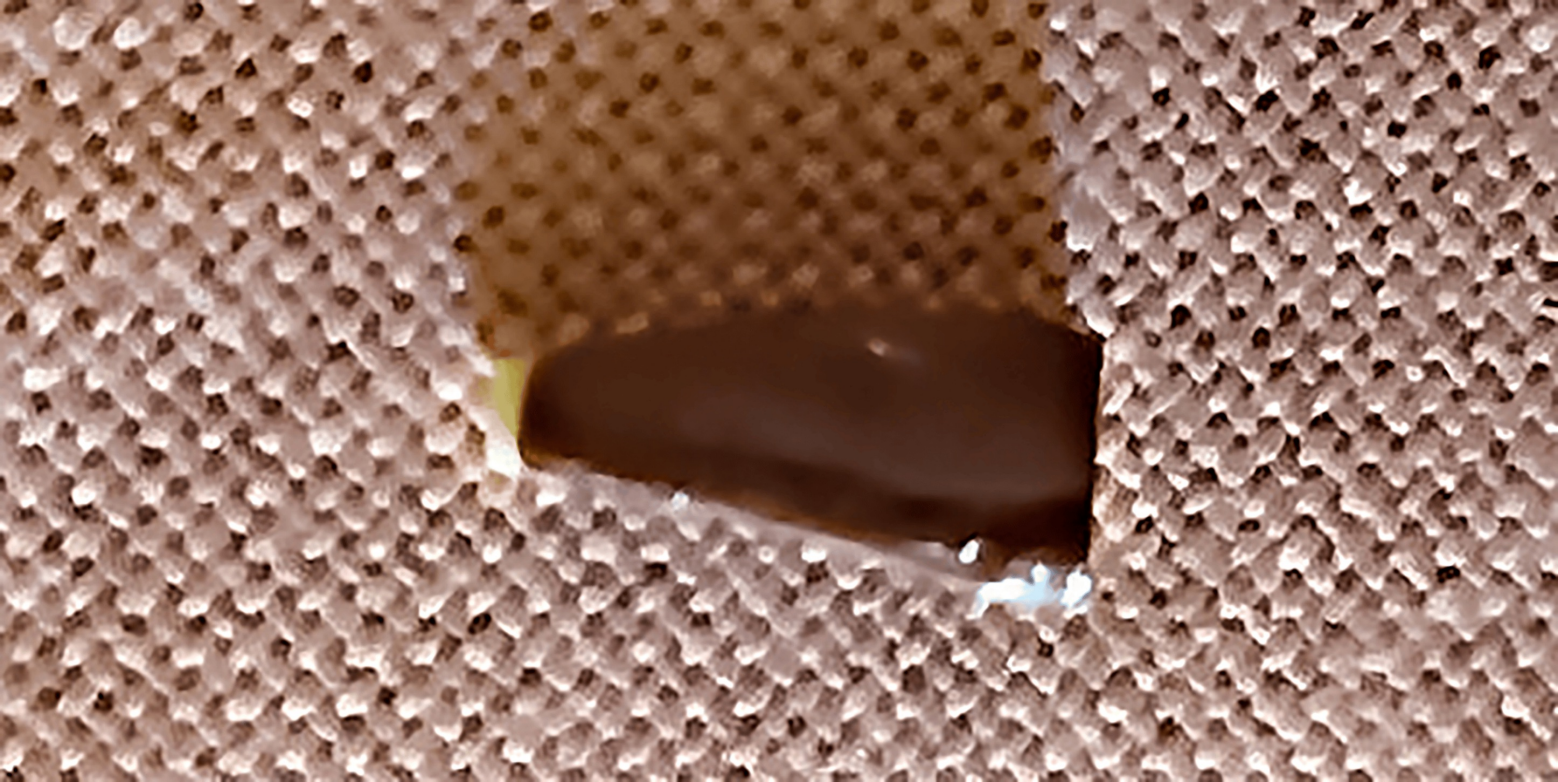
Varnish applied to the tooth leaving the apical 1 mm.

Following the completion of the retrograde filling procedure, each tooth specimen was maintained in an incubator for a duration of 24 hours at a controlled temperature of 37°C and 100% relative humidity. Each tooth was enveloped with a moist cotton pad to ensure optimal conditions. Subsequently, two layers of polishing varnish were meticulously applied, leaving the apical third of each specimen uncovered for the final 1 mm (Figure 6).

**Figure 7:**
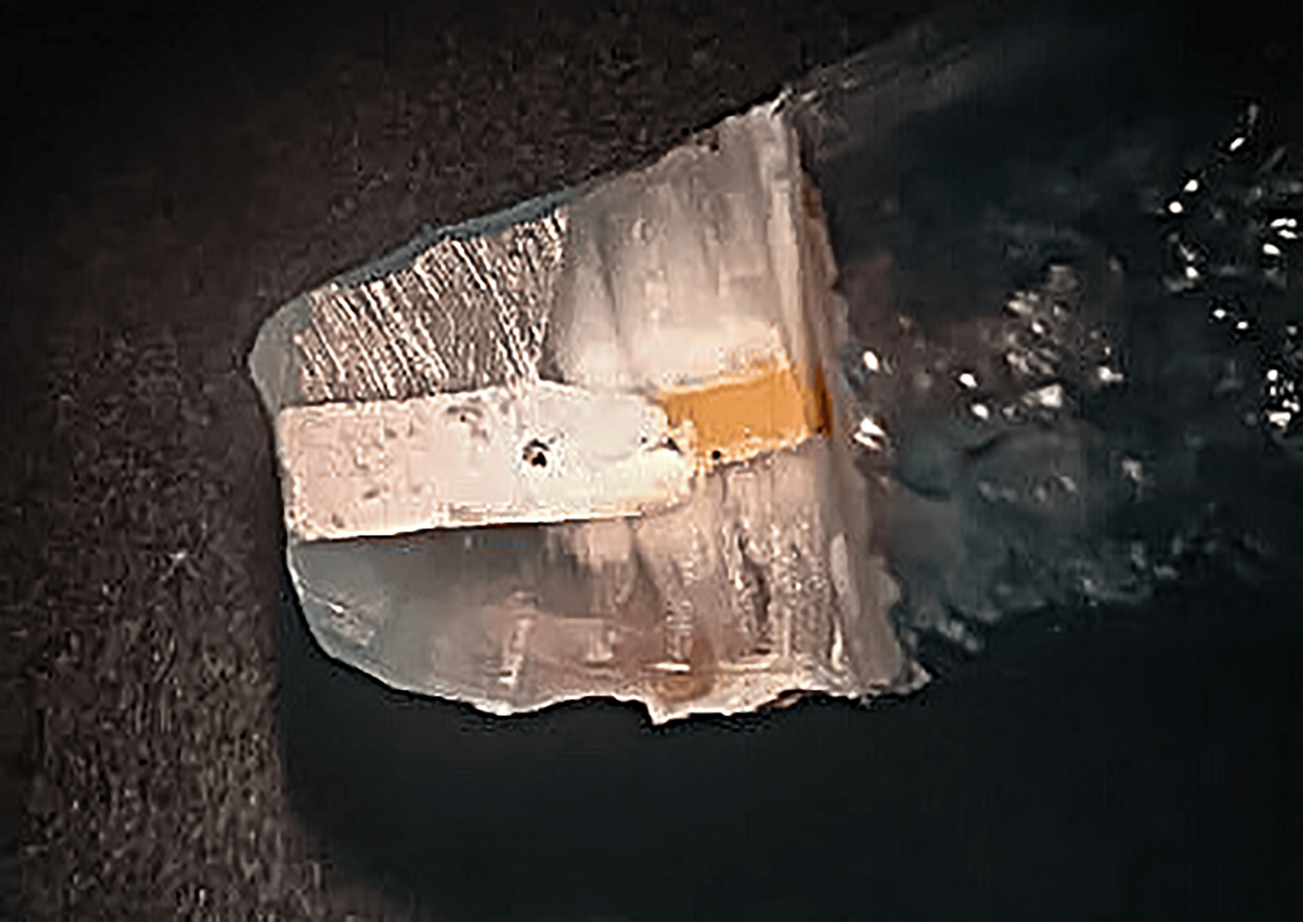
Image of dye penetration in a sectioned tooth taken under a stereomicroscope.

Each tooth was then immersed in a 2% methylene blue dye solution for 36 hours, rinsed with water, and subsequently dried. Thereafter, a longitudinal section of each tooth was prepared using a disc attached to a low-speed handpiece (Marathon-III, Shanghai, China). The specimens were examined under a stereomicroscope at a magnification of 20x to assess the degree of microleakage (Figure 7). The following grading scale was suggested by the authors and used for the evaluation of microleakage: grade 0 = no microleakage; grade 1 = microleakage less than 1 mm; grade 2 = microleakage equal to or greater than 1 mm but less than 2 mm; grade 3 = microleakage equal to or greater than 2 mm.

Statistical analysis

Statistical analysis was performed using SPSS software version 25 (IBM Corp., Armonk, NY). The statistical significance of the difference was tested using the Mann-Whitney U test. The differences were considered significant when p-value ≤ 0.05.

## Results

The depth of penetrated dye in each group (measured in millimeters from the apical end toward the cervical end) is shown in Table 1. The penetration ranged from 0 to 1.6 mm in the Guttaflow Bioseal group (group I) and from 0 to 2.4 mm in the MTA group (group II).

**Table 1:** The depth of dye penetrated in each group measured in millimeters from the apical end toward the cervical end. Values are presented as distance (mm). GFB: Guttaflow Bioseal; MTA: mineral trioxide aggregate.

Tooth number	Group I (GFB)	Group II (MTA)
1	0 mm	0.20 mm
2	0 mm	0 mm
3	0.40 mm	1.60 mm
4	0 mm	0 mm
5	0.6 mm	2.40 mm
6	1.30 mm	2.20 mm
7	0 mm	0 mm
8	0 mm	1.50 mm
9	0.20 mm	0.70 mm
10	0.40 mm	0.50 mm

Table 2 represents the percentage of apical leakage in both groups according to the degree of microleakage.

**Table 2:** Percentages of microleakage in both groups. Values are presented as frequency count (n) and percentage (%).

Root end filling material	Degree of microleakage			
	No microleakage	Less than 1 mm	1-2 mm	More than 2 mm
Guttaflow Bioseal	N = 5 (50%)	N = 4 (40%)	N = 1 (10%)	N = 0 (0%)
Mineral trioxide aggregate (MTA)	N = 3 (30%)	N = 3 (30%)	N = 2 (20%)	N = 2 (20%)

The Mann-Whitney U test results showed that there was no statistically significant difference in the degree of microleakage between the Guttaflow Bioseal group (group I) and the MTA group (group II) (p-value > 0.05), as shown in Table 3.

**Table 3:** Mann-Whitney U test to study the difference in leakage degrees between the two root end filling materials. NS: not significant.

Root end filling material	Rank average	P-value	Significance of differences
Guttaflow Bioseal	8.75	0.19	NS
Mineral trioxide aggregate (MTA)	12.25		

## Discussion

The significance of a tight apical seal in preventing periapical leakage has been underscored by numerous researchers within the field of endodontics. Consequently, extensive studies have been conducted to evaluate the materials employed for retrograde filling during endodontic surgery, focusing on their success and failure rates as well as their efficacy in establishing an apical seal that effectively interrupts the connection between the endodontic canal and periapical tissue. This research contributes to a broader investigation aimed at identifying the most effective material for retrograde filling that achieves optimal apical sealing properties. Specifically, it compares the widely used MTA with Guttaflow Bioseal, a material that has not been previously applied for this purpose in the literature. The study sample comprises single-rooted, single-canal, straight human teeth, which facilitates the control of variables, given that these specimens possess a single apical foramen at most [8-12].

In the present study, the authors investigated a sample comprising 20 mandibular premolars characterized by a single root and single canal. The crowns were sectioned at the level of the cemento-enamel junction. For root canal preparation, a rotary system was employed to ensure the endodontic canals were prepared as uniformly as possible. This approach was chosen due to its status as the most contemporary method within the field of endodontics. For irrigation purposes, sodium hypochlorite at a concentration of 5.25% was utilized. Following this, a final rinse with 17% EDTA was performed.

Due to the significant prevalence of apical ramifications and lateral canals within the final 3 mm of the root structure, a section was performed 3 mm away from the apex of each tooth. This section was executed at a right angle (90 degrees) to the long axis of the tooth. This methodological approach minimizes the exposure of dentinal tubules, thereby facilitating better accessibility to the comprehensive periapical anatomy. Previous studies have demonstrated that performing a vertical cut 3 mm from the apex effectively removes the maximum number of root bifurcations while preserving root length and minimizing the exposure of dentinal tubules. Following the apical cut, retrograde preparation is conducted using ultrasonic tips, which offer several advantages. This technique allows for deeper and more conservative retrograde drilling that adheres to the original trajectory of the root canal, thereby optimizing the positioning of the retrograde preparation and minimizing the risk of lateral perforation. Additionally, this method eliminates the necessity for chamfering the root end to facilitate surgical access, leading to a further reduction in the exposure of dentinal tubules and a decrease in apical leakage [13-16].

Numerous scientific studies have investigated apical sealing, employing a variety of methodologies to assess apical leakage. Among these methods, scanning electron microscopy, radioactive isotopes, electrochemical techniques, and dye penetration studies have been implemented. The use of dyes represents one of the earliest approaches to studying leakage in this context. A range of dye materials has been utilized, including Indian ink, methylene blue, erythrosine solution, and aqueous fuchsin, among others. Each of these substances offers distinct advantages and limitations in evaluating the efficacy of apical sealing [17].

Research examining the efficacy of apical seal ability has often employed pigments, which, despite facing certain criticisms, represent a superior methodological choice. Specifically, dyes are favored over radioisotopes for assessing leakage due to several advantages. Dyes are not only more cost-effective but also safer for both researchers and participants. Furthermore, they are more widely accessible and simpler to handle in experimental settings [17,18]. In the present study, the authors investigated the apical sealing ability of the materials under examination through the application of the methylene blue dye leakage method. This methodology has been widely utilized in prior research to assess the efficacy of various sealing materials [19,20].

To assess the extent of apical leakage, the authors conducted longitudinal sections of the examined samples, as this approach provides a comprehensive perspective of the entire filling. This methodology has been adopted by numerous researchers in the field of endodontics. By utilizing longitudinal sections, a singular view of the filling is achieved, which stands in contrast to the horizontal sectioning method that necessitates multiple sections to adequately evaluate leakage. Thus, this technique facilitates a more thorough examination of apical leakage within a single section, as opposed to the horizontal method, which may compromise the clarity of the findings due to the need for multiple sections [21].

MTA is widely recognized as one of the most pivotal materials utilized in root-end filling procedures. Composed of tricalcium silicate, tricalcium oxide, tricalcium aluminate, silicon dioxide, and bismuth oxide, MTA exhibits several favorable characteristics. Notably, it has a relatively extended setting time of approximately two and a half hours, along with commendable physical properties and exceptional biocompatibility, achieving a pH of around 12.5 within three hours post-preparation [22]. Due to its effective sealing capabilities, MTA has garnered extensive application in root-end filling, ultimately establishing itself as the gold standard for retrograde filling procedures [23].

Recently, Guttaflow Bioseal has been introduced as an innovative material that integrates the functionalities of a sealer with those of gutta-percha, thereby enhancing the sealing efficacy. This product is formulated with finely ground gutta-percha powder, incorporated within a matrix of polydimethylsiloxane and silver nanoparticles [24]. Furthermore, Guttaflow Bioseal contains bioactive components that are capable of releasing restorative factors, which are beneficial for the regeneration and repair of periapical tissues [6,25].

Guttaflow Bioseal represents a significant advancement in endodontic materials by leveraging the beneficial properties of thermoplastic gutta-percha [26]. This biocompatible material is eugenol-free, radiopaque, and exhibits efficient flow characteristics, allowing it to penetrate secondary canals and dentinal tubules effectively. Importantly, Guttaflow Bioseal demonstrates dimensional stability, characterized by a slight volumetric expansion of 0.2% [24,26,27]. The material exhibits high bioactivity due to its incorporation of small bioglass particles, which facilitate the formation of hydroxyapatite crystals or promote their nucleation. Additionally, Guttaflow Bioseal adheres well to dentin, with a working time of five minutes and a setting time ranging from 12 to 16 minutes. These attributes align closely with the ideal requirements of root-end filling materials. Comparative studies indicate that when Guttaflow Bioseal is utilized as a root-end filling material, it exhibits minimal apical leakage and achieves an apical seal comparable to that of MTA.

The effective sealability of Guttaflow Bioseal can be attributed to several key factors, including its strong adhesion to dentin, its notable fluid dynamics that facilitate flow within dentinal tubules, and the minimal volumetric expansion resulting from its substantial water absorption capacity [28]. According to Tanomaru-Filho, Guttaflow Bioseal exhibited an expansion of 0.14% following seven days of storage in distilled water and an expansion of 2.1% after a duration of 30 days. These characteristics contribute to the material's efficacy in achieving a reliable seal in dental applications [25].

While there are no existing studies that utilize Guttaflow Bioseal specifically as a retrograde filling material, research involving its application in endodontic therapy has demonstrated its considerable efficacy in achieving apical sealing [29]. Furthermore, it has been reported that the cytotoxicity of AH Plus exceeds that of Bioseal Guttaflow when evaluated for a long period of time [30].

In addition, a comparative study conducted by Dastorani et al. [30] evaluated bacterial microleakage among various bioactive endodontic filling materials. The findings indicated that Guttaflow Bioseal exhibited superior apical sealing capabilities compared to nano-MTA. Furthermore, the study revealed a high degree of similarity between the two materials regarding their cytotoxicity, highlighting their comparable biocompatibility.

Limitations of the current study

While the study provides valuable insights into the field of retrograde filling materials, several limitations warrant consideration. Firstly, the small sample size may limit the generalizability of the findings. Secondly, the examination was conducted under controlled laboratory conditions, which may not fully replicate the complexities and variabilities encountered in clinical settings. Finally, the duration of incubation and the method of preparation may influence the results; variations in these protocols could yield different outcomes. Future research should aim to address these limitations by incorporating a larger sample size, utilizing multiple assessment methods, and considering different clinical variables to provide a more thorough understanding of the sealing efficacy of these materials.

## Conclusions

In the context of this study, the results demonstrated that there was no statistically significant difference in the efficacy of Guttaflow Bioseal compared to MTA as root-end filling materials following ultrasonic preparation of the root end. The depth of penetrated dye in the Guttaflow Bioseal group was less than that in the MTA group, but it was not statistically significant.

The findings of the current study suggest that Guttaflow Bioseal is a viable option for use as a root-end filling material, as the apical seal achieved with Guttaflow Bioseal was comparable to that achieved using MTA, which is recognized as the gold standard in retrograde filling materials.

## References

[1] Jokinen MA, Kotilainen R, Poikkeus P, Poikkeus R, Sarkki L (1978). Clinical and radiographic study of pulpectomy and root canal therapy. Scand J Dent Res.

[2] Saunders WP, Saunders EM (1994). Coronal leakage as a cause of failure in root-canal therapy: a review. Endod Dent Traumatol.

[3] Virdee SS, Thomas MB (2017). A practitioner's guide to gutta-percha removal during endodontic retreatment. Br Dent J.

[4] Torabinejad M, Higa RK, McKendry DJ, Pitt Ford TR (1994). Dye leakage of four root end filling materials: effects of blood contamination. J Endod.

[5] Gartner AH, Dorn SO (1992). Advances in endodontic surgery. Dent Clin North Am.

[6] Saygili G, Saygili S, Tuglu I, Davut Capar I (2017). In vitro cytotoxicity of GuttaFlow Bioseal, GuttaFlow 2, AH-Plus and MTA Fillapex. Iran Endod J.

[7] Collado-González M, García-Bernal D, Oñate-Sánchez RE (2017). Biocompatibility of three new calcium silicate-based endodontic sealers on human periodontal ligament stem cells. Int Endod J.

[8] Almeida JF, Gomes BP, Ferraz CC, Souza-Filho FJ, Zaia AA (2007). Filling of artificial lateral canals and microleakage and flow of five endodontic sealers. Int Endod J.

[9] De Melo TAF, Nunes DP, Al-Alam FCM, Salles AA, Soares RG (2014). Filling analysis of artificial lateral canals after main canal obturation through three different endodontic sealers. RSBO (Online).

[10] Rodrigues CT, Hussne RP, Nishiyama CK, de Moraes FG (2012). Filling of simulated lateral canals using different obturation techniques: analysis through IDA digital radiograph system. RSBO (Online).

[11] Sonu KR, Girish TN, Ponnappa KC, Kishan KV, Thameem PK (2016). “Comparative evaluation of dentinal penetration of three different endodontic sealers with and without smear layer removal” - scanning electron microscopic study. Saudi Endod J.

[12] Tanomaru-Filho M, Sant'Anna A Jr, Berbert FL, Bosso R, Guerreiro-Tanomaru JM (2012). Ability of gutta-percha and Resilon to fill simulated lateral canals by using the Obtura II system. J Endod.

[13] Amagasa T, Nagase M, Sato T, Shioda S (1989). Apicoectomy with retrograde gutta-percha root filling. Oral Surg Oral Med Oral Pathol.

[14] Rud J, Andreasen JO, Jensen JE (1972). A follow-up study of 1,000 cases treated by endodontic surgery. Int J Oral Surg.

[15] Tidmarsh BG, Arrowsmith MG (1989). Dentinal tubules at the root ends of apicected teeth: a scanning electron microscopic study. Int Endod J.

[16] Vertucci FJ, Beatty RG (1986). Apical leakage associated with retrofilling techniques: a dye study. J Endod.

[17] Erkut S, Tanyel RC, Kekli̇koğlu N, Yildirim S, Kati̇boğlu AB (2006). A comparative microleakage study of retrograde filling materials. Turk J Med Sci.

[18] Youngson CC, Jones JC, Manogue M, Smith IS (1998). In vitro dentinal penetration by tracers used in microleakage studies. Int Endod J.

[19] Ford TR, Torabinejad M, McKendry DJ, Hong CU, Kariyawasam SP (1995). Use of mineral trioxide aggregate for repair of furcal perforations. Oral Surg Oral Med Oral Pathol Oral Radiol Endod.

[20] Lee SJ, Monsef M, Torabinejad M (1993). Sealing ability of a mineral trioxide aggregate for repair of lateral root perforations. J Endod.

[21] Limkangwalmongkol S, Abbott PV, Sandler AB (1992). Apical dye penetration with four root canal sealers and gutta-percha using longitudinal sectioning. J Endod.

[22] Torabinejad M, Ford TR, Abedi HR, Kariyawasam SP, Tang HM (1998). Tissue reaction to implanted root-end filling materials in the tibia and mandible of guinea pigs. J Endod.

[23] Paños-Crespo A, Sánchez-Torres A, Gay-Escoda C (2021). Retrograde filling material in periapical surgery: a systematic review. Med Oral Patol Oral Cir Bucal.

[24] Vasiliadis L, Kodonas K, Economides N, Gogos C, Stavrianos C (2010). Short- and long-term sealing ability of Gutta-flow and AH-Plus using an ex vivo fluid transport model. Int Endod J.

[25] Camargo RV, Silva-Sousa YT, Rosa RP, Mazzi-Chaves JF, Lopes FC, Steier L, Sousa-Neto MD (2017). Evaluation of the physicochemical properties of silicone- and epoxy resin-based root canal sealers. Braz Oral Res.

[26] Varun K, Harpreet S, Rajinder B, Samrity P (2013). Qualitative and quantitative comparative evaluation of sealing ability of Guttaflow, thermoplasticized gutta percha and lateral compaction for root canal obturation: a cohort, controlled, ex-vivo study. Oral Health Dent Manag.

[27] Amanda B, Suprastiwi E, Usman M (2018). Comparison of apical leakage in root canal obturation using bioceramic and polydimethylsiloxane sealer (in vitro). Open J Stomatol.

[28] Gandolfi MG, Siboni F, Prati C (2016). Properties of a novel polysiloxane-guttapercha calcium silicate-bioglass-containing root canal sealer. Dent Mater.

[29] Lee SH, Oh S, Al-Ghamdi AS, Mandorah AO, Kum KY, Chang SW (2020). Sealing ability of AH Plus and GuttaFlow Bioseal. Bioinorg Chem Appl.

[30] Dastorani M, Malekpour B, AminSobhani M, Alemrajabi M, Mahdian A, Malekpour B (2021). Comparison of bacterial microleakage of three bioactive endodontic sealers in simulated underwater diving and aviation conditions. BMC Oral Health.

